# Non-specific complaints at emergency department presentation result in unclear diagnoses and lengthened hospitalization: a prospective observational study

**DOI:** 10.1186/s13049-018-0526-x

**Published:** 2018-07-16

**Authors:** Thomas C. Sauter, Giuliana Capaldo, Michele Hoffmann, Tanja Birrenbach, Stefanie C. Hautz, Juliana E. Kämmer, Aristomenis K. Exadaktylos, Wolf E. Hautz

**Affiliations:** 1 Department of Emergency Medicine, Inselspital, Bern University Hospital, University of Bern, Bern, Switzerland; 2 Department of General Internal Medicine, Inselspital, Bern University Hospital, University of Bern, Bern, Switzerland; 3 Center for Adaptive Rationality, Max Planck Institute for Human Development, Berlin, Germany; 4 AG Progress Test Medizin, Charité Universitätsmedizin, Berlin, Germany

**Keywords:** Non-specific complaints, Emergency department, Length of stay

## Abstract

**Background:**

Up to 20% of patients admitted to an emergency department present with non-specific complaints. Retrospective studies suggest an increased risk of misdiagnosis and hospital admission for these patients, but prospective comparisons of the outcomes of emergency patients with non-specific complaints versus specific complaints are lacking.

**Methods:**

All consecutive patients ≥18 years of age admitted to any internal medicine ward at Bern University Hospital via the emergency department from August 15th 2015 to December 7th 2015 were prospectively included and followed up upon. Patients with non-specific complaints were compared against those with specific complaints regarding the quality of their emergency department diagnosis, length of hospital stay and in-hospital mortality.

**Results:**

Seven hundred and-eleven patients, 165 (23.21%) with non-specific complaints and 546 with specific complaints, were included in this study. No differences between patient groups regarding age, gender or initial severity of the medical problem (deducted from triage category and treatment in a resuscitation bay) were found. Patients with non-specific complaints received more unspecific diagnoses (30.3% vs. 23.1%, *p* = 0.001, OR = 1.82 [95% CI 1.159–2.899]), were hospitalized significantly longer (Median = 6.51 (IQR = 5.85) vs. 5.22 (5.83) days, *p* = 0.025, d = 0.2) but did not have a higher mortality than patients with specific complaints (7.3% vs. 3.7%, *p* = 0.087, OR 1.922 [95% CI 0.909–4.065]).

**Conclusions:**

Non-specific complaints in patients admitted to an emergency department result in low-quality diagnoses and lengthened hospitalization, despite the patients being comparable to patients with specific complaints at admission.

## Background

Up to 20% of patients admitted to any emergency department (ED) present with non-specific chief complaints (NSC), such as generalized weakness, gait disturbance or tiredness [1]. The analysis of a patient’s symptoms is, however, one important cue in diagnosing the patient [1, 2]. Thus, patients presenting with NSC are at an increased risk of misdiagnosis [3]. Indeed, while one out of ten diagnoses is suspected to be incorrect in the general ED population [4, 5], the rate of incorrect ED diagnoses in patients with NSC was found to be as high as 50% [3]. An acute and distinct medical problem requiring emergency medical treatment can ultimately be identified in as much as 51–59% of these patients [6–8], despite the initially unspecific presentation, that may confuse, mislead or even discourage the treating ED team. Consequently, patients with NSC are at an increased risk of non-favorable outcomes [3, 9, 10] such as an increased likelihood of hospital admission [11] and an increased 30-days mortality [1]. Similarly, Djärva and colleagues also describe a four-fold risk of in-hospital mortality for patients with NSC in their retrospective cohort study from Sweden [11]. Most studies on the issue, however, demonstrate that NSC are much more prevalent in elderly patients [8, 11, 12] which makes it difficult to tell whether the unfavorable outcomes of patients presenting with NSC to an ED truly result from their challenging non-specific chief complaint or are simply a consequence of the patients’ older age. Because elderly patients represent the fastest growing emergency department population [8, 12], NSC are consequently of increasing importance to emergency care.

In summary, patients with NSC are not only personally at higher risk of misdiagnosis, hospital admission or even death, but also present a relevant burden to any medical system. Yet, little is known about differences in presentation, diagnostic workup and outcome [13] as prospective comparisons of the outcome of ED patients with NSC versus specific complaints (SC) are lacking. We therefore address the following research questions in a prospective observational study:

1. Do patients with NSC differ from patients with SC in their demographic characteristics and their diagnostic workup in the ED? Based on the literature presented above, we hypothesized that patients with NSC would spend more time in the ED and receive less informative diagnoses.
2. Is there a difference between patients presenting with NSC and patients with SC regarding outcome, measured as length of hospital stay and in-hospital mortality? We hypothesized that patients with NSC would stay longer in hospital and have a higher mortality.

## Methods

The study protocol for this study has been previously published [14].

### Study setting

The data for our prospective observational study were collected at the ED of the Bern University Hospital. The ED is a self-contained, interdisciplinary unit that employs around 45 physicians and 120 nurses, and sees more than 40,000 patients per year, of which around 30% are admitted to the hospital [15]. The department of internal medicine is treating about 4100 cases per year, resulting in about 35,000 hospital days [16].

### Inclusion/exclusion

**Fig. 1:**
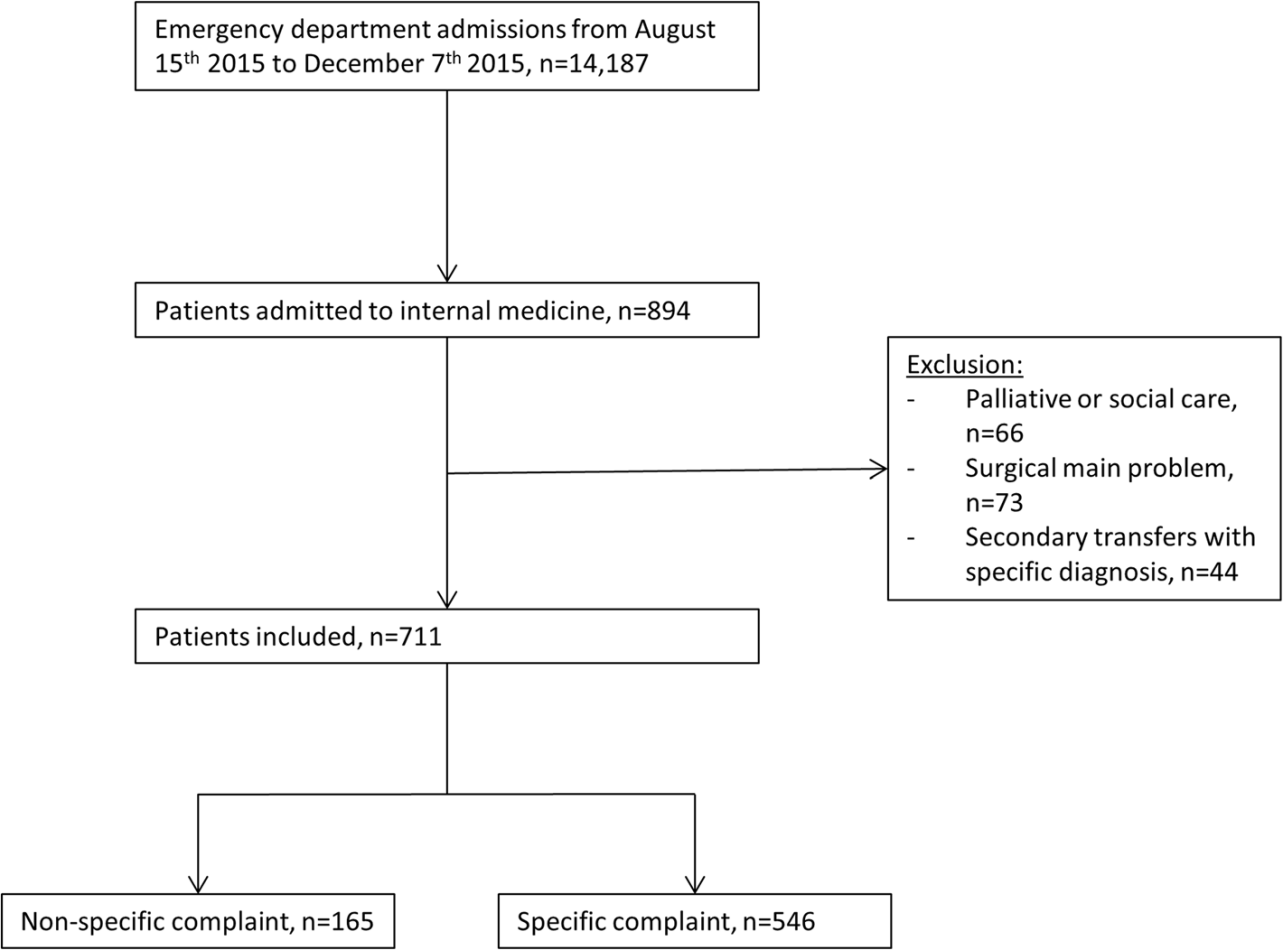
Flowchart

An overview of the inclusion and exclusion procedure is presented in Fig. 1.

We included all patients who were

- 18 years of age or older
  AND
- presented to the emergency department at Bern University Hospital
  AND
- were admitted to any internal medicine (IM) ward at Bern University Hospital.

Patients were excluded if they were

- being admitted to the IM ward for palliative or social care as primary purpose
  OR
- presenting with surgical main problems (e.g. fractures) and admitted to the IM ward because of age or comorbidities
  OR
- were secondarily transferred to our tertiary hospital with a specific external diagnosis, because we expected diagnostic workup of patients fulfilling any of the above exclusion criteria to be substantially different from normal workup.

Patient recruitment lasted from August 15th 2015 to December 7th 2015, which is shorter than the initially planned 9 month [14]. We calculated a sample size of 500 in the study protocol and expected to recruit on average 2 patients per day [14]. We conducted sample size and data checks at predefined time points and found that recruitment worked much faster than expected. Inclusion was consequently stopped for financial reasons after 4 month, resulting in a final sample size of 711.

### Measurements/data

*Patient characteristics* (age, gender), *encounter characteristics* (initial complaint at admission, previous medical contacts (general practitioner, ambulance services), triage category, treatment in a resuscitation bay) and *outcome data* (length of ED stay, length of hospital stay, ED and IM discharge diagnosis, in-hospital mortality) were prospectively recorded using data routinely documented in the clinical information system (E-Care, ED 2.1.3.0, Turnhout Belgium) by the responsible medical treatment team.

In our ED, all admitted ED patients are triaged by registered nurses using the Swiss triage scale [17], ranging from 1 (acute situation, immediate care) to 4 (non-urgent situation) and including a fifth level (scheduled visit; see Table 1). Both, triage category at ED admission and the decision of the ED team to treat the patient in the resuscitation bay can serve as surrogate parameters of the medical severity of a patient’s presentation.

**Table 1:** Comparison of patient groups with non-specific complaints (NSC) (*n* = 165) or specific complaints (SC) (*n* = 546)

Variable	NSC (*n* = 165)	SC (*n* = 546)	*p*-value
Patient characteristics			
Age in years; median (IQR)	71.0 (23.0)	69.0 (27)	0.347
Female gender; n (%)	79 (47.9%)	243 (44.5%)	0.476
Encounter characteristics			
Resuscitation bay; n (%)	14 (8.5%)	64 (11.8%)	0.259
Triage category; n (%)			
1 (acute situation, immediate care)	10 (6.1%)	32 (5.9%)	0.282
2 (urgent situation, care within 20 min)	58 (35.2%)	241 (44.1%)	
3 (semi-urgent situation, care within 120 min)	89 (53.9%)	255 (46.7%)	
4 (non-urgent situation)	4 (4.2%)	14 (2.6%)	
5 (scheduled visit)	1 (0.6%)	4 (0.7%)	
Previous medical contact; n (%)	162 (67.3%)	289 (52.9%)	< 0.001
ED outcome			
ED stay in hours; median (IQR)^a^	6.27 (3.11)	6.09 (3.26)	0.497°
Vague diagnosis at ED discharge; n (%)	50 (30.3%)	126 (23.1%)	0.039°
Patient outcome			
Hospital stay in days; median (IQR) ^b^	6.51 (5.85)	5.22 (5.83)	0.005°
Vague diagnosis at hospital discharge; n (%)	15 (9.1%)	51 (9.3%)	0.531°
Mortality; n (%)	12 (7.3%)	20 (3.7%)	0.045°

*ED* emergency department, *NSC* non-specific complaints, *SC* specific complaint,

°one-sided test according to a priori hypotheses

^a^calculated in minutes and converted to hours

^b^calculated in hours and converted to days

NSC presentations are manifold, often vague and diffuse and therefore difficult to classify [13]. Examples include generalized weakness [18], gait disturbance [19] and tiredness [20]. Nemec and colleagues introduced a list of specific complaints of conditions for which emergency medicine treatment algorithms exist and proposed to classify the absence of any of these complaints as non-specific [1]. Following this approach, two independent raters (GC and MH) classified the presenting complaint of all included patients as either non-specific or specific independently and in duplicate, according to the list published by Nemec. Interrater agreement was calculated as Cohen’s kappa.

All diagnosis collected throughout the study were classified according to the clinical modification of the international classification of diseases, version 10 (ICD-10-CM). Two independent raters (GC and MH) independently and in duplicate classified the diagnoses of 100 randomly selected patients and achieved perfect rater agreement (kappa = 0.96). Each then classified half of the remaining diagnoses alone.

All ICD-10-CM codes were then summarized into diagnostic groups using the clinical classification software (CSS) [21]. The software groups diseases by ICD code into 18 different groups such as “cardio-circulatory diseases” or “infectious diseases”. All diagnoses classified as “residual codes” or “symptoms; signs, ill-defined conditions and factors influencing health status” were summarized as *vague diagnoses*. We would argue that these diagnoses, which include labels such as “generally degraded health status” (ICD code R53) or “fever of unknown origin” (ICD code R50.9) to be of low quality because they have little therapeutic or prognostic value and potentially encompass a large variety of underlying diseases. We compared these *vague diagnoses* against all other CCS groups, which we termed *distinct diagnoses*.

### Data analysis

For statistical analysis, SPSS Statistics (Version 22 (IBM New York, USA) was used.

Baseline characteristics are presented as numbers and percentage for categorical and ordinal variables (gender, presentation through the resuscitation bay, triage, previous medical contact, vague diagnosis at ED or hospital discharge, mortality). For metric variables (age, ED stay, hospital stay), we tested for normality using Shapiro Wilks test. Because all these variables were non-normally distributed, we describe them using Median and interquartile range (IQR). The median for ED stay was calculated in minutes and then converted to hours to facilitate readability. Likewise, the median for length of hospital stay was calculated in hours and converted to days. The group of patients with NSC was compared to those with SC by means of Chi-square tests (nominal variables), and Mann-Whitney-U test (all ordinal and metric variables). For all tests, a *p*-value < 0.05 is considered significant.

To determine the association of each variable with the specificity of the presenting complaint (specific or non-specific), we conducted univariate binary logistic regressions for each variable individually. All variables with a *p* < 0.1 in univariate regression were included into one of two multivariate binary logistic regressions. One multivariate regression was conducted for the variables assumed to be predictive of complaint specificity (age, gender, previous medical contact) and one regression for variables assumed to be a consequence of complaint specificity (triage category, treatment in resuscitation bay, duration of ED and hospital stay, vagueness of diagnosis at ED and hospital discharge, mortality).

### Ethics

The present study is registered and approved by the responsible ethics committee of the Canton Bern, Switzerland (Nr. 197/15) as a quality evaluation study. According to Swiss law, the need to obtain informed consent was waived by the ethics committee.

## Results

During the study period, 14′187 patients presented to the ED of which 894 patients were admitted to an IM ward. Of those admitted, 66 patients were excluded from further analysis because of palliative or social care as main reason for hospital admission, 73 patients because of a surgical main problem and 44 patients because of a secondary transfer with an established diagnosis at ED admission (Fig. 1). Out of the 711 patients finally included in this study, 165 (23.21%) presented with NSC and 546 with SC.

Raters achieved perfect agreement in the classification of chief complaints as either specific or non-specific (kappa = 0.9) and in the assignment of ICD-10-CM codes (kappa = 0.96).

Overall, Patients were on average 65.62 years old, 322 (45.3%) were female, 400 (56.3%) had previous medical contacts and 78 (11%) were seen in a resuscitation bay. A comparison of patients presenting with NSC versus those with SC is presented in Table 1.

No difference between patient groups regarding age, gender or initial impression of severity of the medical problem (as indicated by triage category and treatment in resuscitation bay) was found. The patients with NSC, however, had significantly more previous medical contacts (67.3% vs. 52.9% patients, *p* < 0.001).

Regarding ED outcome, patients with NSC did not stay longer in the ED compared to those with SC (NSC Median = 6.27 (IQR = 3.11) versus SC 6.09 (3.26), *p* = 0.497). The rate of vague diagnosis at ED discharge was significantly higher in the patient group with NSC at ED admission (30.5% vs. 23.1%, *p* = 0.039). At hospital discharge, the difference in the quality of their diagnosis was no longer significant between patients presenting with NSC vs. those with SC (9.1% vs. 9.3%, *p* = 0.531).

Patients with NSC stayed significantly longer in hospital (Median = 6.51 (IQR = 5.85) versus 5.22 (5.83) days, *p* = 0.005) and had a higher mortality compared to patients in the specific complaint group (12 (7.3%) vs. 20 (7.3%), *p* = 0.045). This effect is however observable in one-sided testing according to the prior hypothesis.

Of the variables assumed to be predictive of complaint specificity, only a previous medical contact was significantly associated with complaint specificity in univariate logistic regression (Table 2). Of the variables assumed to be a consequence of complaint specificity, length of hospital stay, quality of the ED diagnosis and mortality were associated on a significance level of *p* < 0.1 with complaint specificity in univariate regression (Table 2). The association with complaint specificity held for all variables except for mortality in multivariate regression (Table 2) on a significance level of *p* < 0.05.

**Table 2:** Results of logistic regression of variables assumed to be predictive or a consequence of complaint specificity

Factor	*p* in univariate regression^*^	*p* in multivariate regression ^°^	Odds ratio in multivariate regression [95% CI]
Predictive of complaint specificity			
Age	0.171		
Gender	0.466		
Previous medical contact	**0.001**	**0.001**	1.828 [1.268–2.636]
Consequences of complaint specificity			
Triage	0.101		
Resuscitation bay	0.241		
Length of ED stay	0.748		
Vague diagnosis at ED discharge	**0.012**	**0.001**	1.822 [1.159–2.899]
Length of hospital stay	**0.029**	**0.025**	1.001 [1–1.002]
Vague diagnosis at ED discharge	0.923		
Mortality	**0.054**	0.087	1.922 [0.909–4.065]

^*^significance defined as *p* < 0.1

°significance defined as *p* < 0.05 (see methods section for details)

## Discussion

Non-specific chief complaints are frequent among patients presenting to any emergency department [1]. In this prospective observational study of emergency patients hospitalized to internal medicine wards at a single center, 23.3% presented with non-specific complains. These patients present a diagnostic challenge compared to those with specific complaints [3, 9], indicated by their significantly higher likelihood of previous medical contacts prior to emergency presentation and the low quality of diagnoses received at the emergency room. This finding corresponds well to the results of previous retrospective studies. For example, Peng and colleagues found that in more than half of the patients presenting to their emergency room, the EDs diagnosis was overruled within the next 90 days [4], indicating ED diagnoses of low quality.

Unspecific complaints at presentation may confuse, mislead or even discourage the treating ED team [6–8], and no guidelines for the diagnostic workup of patients presenting with NSC are currently available [7]. Indeed, diagnostic uncertainty has previously been termed the “hallmark of emergency medicine, [given that] emergency physicians are challenged daily by the vast spectrum and acuity of clinical presentations they diagnose in a data-poor, rapidly evolving, decision-dense environment” [22]. Consequently, particular attention should be paid to patients presenting with (seemingly non-urgent) non-specific complaints.

One consequence of an emergency presentation with NSC is their higher likelihood to receive a descriptive or otherwise low-quality diagnosis in the emergency room. This has to be seen in the context of the fact, that an acute and distinct medical problem requiring emergency medical treatment can ultimately be identified in as much as 51–59% of NSC patients [6–8]. The need to further develop and improve the low-quality diagnosis of the ED is evident in our study by the significantly longer time patients with non-specific complaints are hospitalized (1 day on average), although the effect is small. At hospital discharge, patients with non-specific complaints do not differ anymore from those with specific complaints with regard to the quality of their diagnosis. A previous Danish study similarly identified frail patients, a group that often presents with non-specific complaints, to be at an increased risk for admission, length of stay and even mortality after visits to the ED [23]. Given the size of the affected patient population and the financial implications of lengthened hospitalization, this finding further emphasizes the need for an increased attention to patients presenting with non-specific complaints. A systematic review of scoring systems recently compared ten different strategies for risk stratification at ED admission and also emphasized the need for more research, as none of the systems predicted all important outcomes with high precision [24]. Possible approaches include screening for “immobility as a vital sign” [25], reassessing patients with low-quality and descriptive diagnoses [23] or the algorithmic implementation of predefined diagnostic packages triggered by chief complaints [26].

Interestingly, patients presenting with non-specific complaints in our study were neither older nor triaged more urgently at ED admission. This lack of differences at presentation between the groups of patients with NSC and SC stands in contrast to previous studies that report an increased number of female and older patients to present with NSC [2, 9, 13, 27]. This discrepancy may result from the fact that we, by choice, only looked at a rather homogeneous population of patients hospitalized to an internal medicine ward. We deliberately limited our investigation to hospitalized ED patients for three reasons: First, we assumed adverse outcomes to be more frequent in hospitalized patients as compared of those discharged from the ED, thus increasing the statistical power of the study. Second, we suspected the age-distribution of hospitalized patients presenting with SC to be comparable to hospitalized patients with NSC, because the risk for hospitalization generally increases with age, regardless of the presenting complaint. Because NSC are more prevalent in an elderly population, we aimed to establish a similarly old comparison group by including only hospitalized patients. Third, the burden on the health care system is larger in hospitalized than in discharged patients, thus increasing the relevance of any potential finding of this study.

A last finding in our study is the observation that although patients with non-specific complaints have a substantially higher in-hospital mortality than those without (7.5% vs. 3.7%), in-hospital mortality is not a consequence significantly associated with complaint specificity in multivariate regression.

Christensen et al. report an in-hospital mortality of 2.38% and a 30-days mortality of 4.32% in NSC patients [23]. Other mortality rates of NSC patients described in previous studies range from a 30-days mortality in Swiss ED patients with NSC of 6% [1] to an in-hospital mortality of 13% in a Swedish ED population [11]. Compared to this mortality rates, the 7.3% in-hospital mortality of NSC patients found in our study population is rather at the lower limit of previously described mortality rates.

### Limitation

Our study has several limitations that warrant discussion. Because it is limited to a single center, the generalizability of our findings to other populations remains unknown. Further multi-centric investigations are necessary to investigate our findings in larger and potentially more diverse populations. In addition, more data about the exact diagnostic workup of NSC patients should be obtained in future investigations, as our dataset cannot provide an exact breakdown of the steps of the diagnostic efforts. For example, the disappearing of the significant difference in the amount of distinct diagnosis from ED discharge until IM ward discharge may be the result of a longer hospital stay. In addition, with our data, it is not possible to break down exactly which aspect of the diagnostic workup or the more complex and time-consuming therapy on the IM ward caused the NSC patients to stay significantly longer. Last, we used one-sided testing to compare single variables between groups. It could be argued that this approach increases the chances to detect significant differences and thus could increase the likelihood of false positive findings. We would argue that the formulation of a priori hypothesis based on the literature on non-specific complaints justifies the use of one-sided testing.

## Conclusion

In conclusion, our study highlights the importance of the ED patients presenting with NSC, as they have an increased risk of unfavorable outcome. One practical implication of the results described above is that each patient presenting with NSC should trigger an awareness in every ED physician, that an unfavorable outcomes is likely. In addition, the development of a specific treatment algorithm for patients with NSC in analogy to existing recommendations for e.g. chest pain or sepsis may be another possible way to improve the outcome in these patients. On the systems level, an adaption of existing triage scales in order to increase attention to patients with NSC may be a fruitful approach for further investigation [13].

## Abbreviations

ED: Emergency department
IM: Internal medicine
NSC: Non-specific complaints
SC: Specific complaints

## References

[1] Nemec M, Koller MT, Nickel CH, Maile S, Winterhalder C, Karrer C (2010). Patients presenting to the emergency department with non-specific complaints: the Basel non-specific complaints (BANC) study. Acad Emerg Med Off J Soc Acad Emerg Med.

[2] Hampton JR, Harrison MJ, Mitchell JR, Prichard JS, Seymour C (1975). Relative contributions of history-taking, physical examination, and laboratory investigation to diagnosis and management of medical outpatients. Br Med J.

[3] Gruppen LD, Woolliscroft JO, Wolf FM (1988). The contribution of different components of the clinical encounter in generating and eliminating diagnostic hypotheses. Res Med Educ.

[4] Peng A, Rohacek M, Ackermann S, Ilsemann-Karakoumis J, Ghanim L, Messmer AS (2015). The proportion of correct diagnoses is low in emergency patients with nonspecific complaints presenting to the emergency department. Swiss Med Wkly.

[5] Saber Tehrani AS, Lee H, Mathews SC, Shore A, Makary MA, Pronovost PJ (2013). 25-year summary of US malpractice claims for diagnostic errors 1986-2010: an analysis from the National Practitioner Data Bank. BMJ Qual Saf.

[6] Committee on Diagnostic Error in Health C, Board on Health Care S. In: Balogh E, Miller B, Ball J, editors. Improving diagnosis in health care: National Academies Press (US); 2015. 26803862

[7] Karakoumis J, Nickel CH, Kirsch M, Rohacek M, Geigy N, Müller B (2015). Emergency presentations with nonspecific complaints-the burden of morbidity and the Spectrum of underlying disease: nonspecific complaints and underlying disease. Medicine (Baltimore).

[8] Rutschmann OT, Chevalley T, Zumwald C, Luthy C, Vermeulen B, Sarasin FP (2005). Pitfalls in the emergency department triage of frail elderly patients without specific complaints. Swiss Med Wkly.

[9] Ruger JP, Lewis LM, Richter CJ (2007). Identifying high-risk patients for triage and resource allocation in the ED. Am J Emerg Med.

[10] Safwenberg U, Terént A, Lind L (2007). The emergency department presenting complaint as predictor of in-hospital fatality. Eur J Emerg Med.

[11] Djärv T, Castrén M, Mårtenson L, Kurland L (2015). Decreased general condition in the emergency department: high in-hospital mortality and a broad range of discharge diagnoses. Eur J Emerg Med.

[12] Samaras N, Chevalley T, Samaras D, Gold G (2010). Older patients in the emergency department: a review. Ann Emerg Med.

[13] Bingisser R, Nickel CH (2013). The last century of symptom-oriented research in emergency presentations--have we made any progress?. Swiss Med Wkly.

[14] Hautz SC, Schuler L, Kämmer JE, Schauber SK, Ricklin ME, Sauter TC (2016). Factors predicting a change in diagnosis in patients hospitalised through the emergency room: a prospective observational study. BMJ Open.

[15] Exadaktylos A, Hautz WE (2015). Emergency medicine in Switzerland. ICU Manag.

[16] Inselgruppe (2016). Jahresbericht Inselgruppe.

[17] Rutschmann OT, Hugli OW, Marti C, Grosgurin O, Geissbuhler A, Kossovsky M, et al. Reliability of the revised Swiss emergency triage scale: a computer simulation study. Eur J Emerg Med Off. 2017; 10.1097/MEJ.0000000000000449 PMC6039392 28099182

[18] Nickel CH, Nemec M, Bingisser R (2009). Weakness as presenting symptom in the emergency department. Swiss Med Wkly.

[19] Newman AB, Gottdiener JS, Mcburnie MA, Hirsch CH, Kop WJ, Tracy R (2001). Associations of subclinical cardiovascular disease with frailty. J Gerontol A Biol Sci Med Sci.

[20] Avlund K, Schultz-Larsen K, Davidsen M (1998). Tiredness in daily activities at age 70 as a predictor of mortality during the next 10 years. J Clin Epidemiol.

[21] HCUP User Support (HCUP-US) RMHCaUPH, Agency for Healthcare Research and Quality (US). Clinical Classifications Software (CCS) for ICD-10-CM/PCS. [cited 2018 May 29]. Available from: https://www.hcup-us.ahrq.gov/toolssoftware/ccs10/ccs10.jsp

[22] Ilgen JS, Humbert AJ, Kuhn G, Hansen ML, Norman GR, Eva KW (2012). Assessing diagnostic reasoning: a consensus statement summarizing theory, practice, and future needs. Acad Emerg Med.

[23] Christensen EF, Larsen TM, Jensen FB, Bendtsen MD, Hansen PA, Johnsen SP (2016). Diagnosis and mortality in prehospital emergency patients transported to hospital: a population-based and registry-based cohort study. BMJ Open.

[24] Brabrand M, Folkestad L, Clausen NG, Knudsen T, Hallas J (2010). Risk scoring systems for adults admitted to the emergency department: a systematic review. Scand J Trauma Resusc Emerg Med.

[25] Brabrand M, Kellett J, Opio M, Cooksley T, Nickel CH. Should impaired mobility on presentation be a vital sign? Acta Anaesthesiol Scand. 2018; 10.1111/aas.13098 29512139

[26] Nørgaard B, Mogensen CB, Teglbjærg LS, Brabrand M, Lassen AT. Diagnostic packages can be assigned accurately in emergency departments. A multi-centre cohort study. Dan Med J. 2016;63(6) 27264941

[27] Policies ECAsraE. The 2015 Ageing Report. [cited 2018 May 29]. Available from: http://www.aal-europe.eu/wp-content/uploads/2015/08/Ageing-Report-2015.pdf

